# Polysulfone-Based Membranes Modified with Ionic Liquids and Silica for Potential Fuel Cell Applications

**DOI:** 10.3390/membranes14120270

**Published:** 2024-12-13

**Authors:** Emma Fernández-Llamazares, Thi Hai Van Nguyen, Pere Verdugo, Aitor Gual, Diogo M. E. Garcia, Claudia Delgado Simão, Miriam Díaz de los Bernardos, Adrianna Nogalska

**Affiliations:** 1 Unit of Chemical Technologies, Technology Centre of Catalonia, Eurecat, 43007 Tarragona, Spain; emma.fernandez@eurecat.org (E.F.-L.); pere.verdugo@eurecat.org (P.V.); aitor.gual@eurecat.org (A.G.); miriam.diaz@eurecat.org (M.D.d.l.B.); 2 Unit of Functional Printing and Embedded Devices, Technology Centre of Catalonia, Eurecat, 08302 Mataró, Spain; van.nguyen@eurecat.org (T.H.V.N.); diogo.garcia@eurecat.org (D.M.E.G.); claudia.delgado@gmail.com (C.D.S.); 3 Department of Analytical and Organic Chemistry, Universitat Rovira i Virgili, 43007 Tarragona, Spain

**Keywords:** proton exchange membrane fuel cells, polysulfone membranes, ionic liquids, polydimethylsiloxane-functionalized silica, proton permeability, proton conductivity, phase inversion method, porosity, hydrophilicity, surface roughness

## Abstract

The urgent need for sustainable, low-emission energy solutions has positioned proton exchange membrane fuel cells (PEMFCs) as a promising technology in clean energy conversion. Polysulfone (PSF) membranes with incorporated ionic liquid (IL) and hydrophobic polydimethylsiloxane-functionalized silica (SiO_2_-PDMS) were developed and characterized for their potential application in PEMFCs. Using a phase inversion method, membranes with various combinations of PSFs, SiO_2_-PDMS, and 1-butyl-3-methylimidazolium triflate (BMI.TfO) (1–10 wt%) were prepared and characterized to assess their morphology, porosity, wettability, ionic conductivity, and thermal stability. Incorporating IL significantly altered the membrane structure, increasing porosity and surface roughness, while SiO_2_-PDMS enhanced IL retention, reducing leakage by up to 32%. Proton conductivity increased by up to 30 times compared to pure PSF, and membranes exhibited high hydrophilicity at optimal IL concentrations. This work highlights the potential of IL and silica-based membranes for practical applications in PEMFCs.

## 1. Introduction

Proton exchange membrane fuel cells (PEMFCs) represent a promising technology for clean energy conversion, addressing the urgent need for sustainable and efficient energy solutions [1,2,3]. The transition toward alternative fuels is crucial in mitigating climate change and reducing reliance on fossil fuels, which are major contributors to greenhouse gas emissions [4,5]. Hydrogen, often recognized as a renewable energy carrier, has the potential to significantly contribute to the decarbonization of energy systems. Among the various hydrogen production methods, water electrolysis powered by renewable energy sources has been gaining considerable attention [6]. This approach addresses the intermittent and variable nature of renewable energy systems, such as wind, solar, and wave power, by improving their stability and facilitating their integration into energy networks [7,8]. Liquid hydrogen carriers, such as alcohols, acids, and sugars, represent an appealing alternative due to their higher energy density and easier transport, storage, and handling. These carriers offer a practical solution to some of the challenges associated with using hydrogen gas, including complex infrastructure requirements and safety concerns [9]. Among liquid carriers, formic acid and methanol have attracted significant attention, as they can be produced from CO_2_ reduction, creating a carbon-neutral fuel cycle [10]. Fuel cells operate by converting chemical energy into electrical energy through electrochemical reactions between the fuel and oxygen from air, producing electricity and water as the main byproduct. This clean energy conversion makes fuel cells an attractive option for a wide range of applications, from powering vehicles to providing stationary energy solutions. Various types of fuel cells exist, including alkaline fuel cells (AFCs), phosphoric acid fuel cells (PAFCs), and solid oxide fuel cells (SOFCs), among others, but this study focuses specifically on PEMFCs due to their operational advantages, such as low-temperature functionality and rapid start-up times [11]. As highlighted by Xu et al. [12], PEMFCs are highly efficient at converting chemical energy directly to electricity, which makes them well suited for reducing greenhouse gas emissions and advancing hydrogen-based energy applications. However, long-term performance degradation remains a significant challenge for commercialization. This degradation arises from a combination of factors, including the deterioration of membrane and electrode materials, catalyst loss, mechanical damage to the membrane electrode assembly (MEA), and fluctuations in operating conditions [13].

At the heart of PEMFC operation is the membrane electrode assembly (MEA), which plays a crucial role in facilitating the electrochemical reactions necessary for energy generation. The MEA consists of a proton exchange membrane (PEM) sandwiched between catalyst layers for the anode and cathode, each covered with gas diffusion layers. The PEM serves a dual purpose; it selectively allows for the transport of protons while functioning as an electrical insulator, which prevents direct electrical shortcuts between the electrodes. This design forces electrons to travel through an external circuit, thus generating usable electrical energy [11]. Falina et al. underscore the important role of membrane characteristics, such as thickness and water content, in enhancing ion transport and lowering resistance within PEMFCs, influencing their energy efficiency and lifespan [14]. It is also important to note that other factors, such as electrode degradation and catalyst loss, also contribute to overall performance decline.

Currently, Nafion^®^ (Chemours) and Aquivion^®^ (Solvay) are the most widely used materials for PEMs, both of which are based on perfluorinated sulfonic acids [15]. The Nafion polymer is structured from perfluorovinyl ether groups terminated with sulfonate groups, all of which are attached to a robust polytetrafluoroethylene (PTFE) backbone. In comparison, Aquivion possesses a similar structure but features shorter side chains, resulting in enhanced crystallinity, superior mechanical properties, and improved proton conductivity. These membranes are favored for their high proton conductivity and excellent mechanical strength, essential qualities for effective fuel cell operation.

Despite the advantages of these conventional membranes, there is a growing interest in exploring alternative polymeric materials to reduce costs while maintaining or even enhancing performance [16,17]. Furthermore, proton conducting within Nafion membranes occurs via the vehicle mechanism, which is typically employed at low temperatures and high moisture levels. In this process, water plays a crucial role by transporting protons across the membrane (through the hydronium form), achieving proton conductivities up to 100 mS/cm at 95 °C (set as a standard by the USA Department of Energy (DOE)) [18]. However, this conductivity decreases at higher temperatures (>100 °C) due to reduced humidification [19]. Higher temperatures are desirable for several reasons, including reduced CO catalyst poisoning and faster kinetics. A particularly promising avenue involves the incorporation of ionic liquid (IL) in PEMFC applications, of which over 200 articles have been published annually in the past decade, including membrane and catalyst modifications [20,21]. Their unique properties, including low volatility and flammability, a wide electrochemical window, and high ionic conductivity, make them ideal candidates for fuel cell applications. IL has been shown to enhance proton conductivity and mitigate challenges associated with water management and thermal stability.

Effective water management is crucial for maximizing the performance and durability of PEMFCs. Typically, high humidity in the inlet gasses is required to maintain membrane hydration and ensure optimal proton conductivity. Moreover, water is generated on the anode as result of an O_2_ and proton reaction. The excessive liquid water can accumulate in the pores of the catalyst layer (CL) and gas diffusion layer (GDL), increasing mass transport resistance. Therefore, it is important to balance membrane hydration and water flooding to prevent fuel cell degradation [22,23]. Operating PEMFCs at temperatures above 100 °C can help address these challenges. At elevated temperatures, humidity decreases and the proton conduction mechanism shifts to the Grotthuss mechanism, where protons are transferred through the formation and reformation of hydrogen bonds. The incorporation of IL into PEMs helps maintain appropriate conductivity, even at higher temperatures [19].

Different ILs have been incorporated into different polymer matrices such as Nafion, poly (vinylidene fluoride-co-hexafluoropropylene) (PVDF–HFP), sulfonated poly (ether ether ketone) (SPEEK), and sulfonated poly (ether ketone) (SPEK) by the solution casting method. Goh et al. demonstrate how the addition of ionic liquids like 2–hydroxyethylammonium formate (2–HEAF) and propylammonium nitrate (PAN) into Nafion^®^ membrane improves (a) proton conductivity, obtaining maximum values of 2.87 and 4.55 mS/cm, respectively, which are 2.2 and 3.5 times higher than that of the pure recast Nafion^®^ membrane and (b) thermal stability, as the addition of ionic liquids improves the thermal resistance of the membrane by forming stable ionic pairs with the sulfonic acid groups in Nafion^®^. This interaction stabilizes the membrane structure and delays the onset of thermal degradation [24].

Gao et al. [25] explore the use of the ionic liquid 1-ethyl-3-methylimidazolium hydrogen sulfate ([Emim] HSO_4_) as an electrolyte in proton exchange membrane fuel cells (PEMFCs) at high temperatures. Results show that [Emim] HSO_4_ improves conductivity as temperature rises, increasing from approximately 0.25 mS/cm at 25 °C to 2.8 mS/cm at 150 °C. Additionally, they report a maximum power density of 0.6 mW/cm^2^ at 168 °C, which may enable PEMFCs to function with reformed fuels by reducing CO poisoning at elevated temperatures. Nair et al. [26] present a novel proton-conductive polymer electrolyte combining perchloric acid-functionalized nano-silica with diethylmethylammonium trifluoromethanesulfonate ([dema] [TfO]) into a PVDF–HFP matrix. Scanning electron microscopy (SEM) images reveal the evolution of surface microporosity with IL concentration. The introduction of nano-silica was aimed at enhancing IL retention. The composite material shows increased ionic conductivity and stability at room and high temperatures, reaching maximum conductivities of 0.02 mS/cm at 30 °C and 0.6 mS/cm at 100 °C for 80 wt% of [dema] [TfO]. However, a critical challenge associated with the use of IL is its tendency to leak from the membrane matrix, which can significantly compromise long-term stability and overall performance [19,20]. Different studies investigated that phenomenon. Malik et al. [27] explored composite membranes made from sulfonated polyetherketone (SPEK) and imidazole-based IL. They compared 1-Butyl-3-methylimidazolium trifluoromethanesulfonate [BMI.TfO] as a hydrophobic IL with 1-Butyl-3-methylimidazolium bis (trifluoromethylsulfonyl)imide (BMI.NTf_2_) as a hydrophilic IL. The best anhydrous ion conductivity was found to be 5 mS/cm at 140 °C for SPEK/(BMI.TfO)-70, being around 100 times higher than a blank SPEK membrane. Furthermore, the study demonstrates that higher IL concentrations and operating temperatures (e.g., 80 °C) led to increased IL leakage. This finding underscores the importance of selecting ILs with low hydrophilicity to minimize leakage under high-temperature PEMFC conditions. Jothi et al. [28] examined composite membranes made from SPEEK combined with 1-Ethyl-3-methylimidazoliumdiethyl phosphate (EMIMDEP). They reported good conductivities at 145 °C, ranging from 1.25 to 3.16 mS/cm. IL leaching studies conducted in a water medium showed that lower concentrations helped reduce leaching during operation. Composite membranes (SPEEK/SiOx/OTf) were synthesized with SPEEK, tetraethoxysilane (TEOS), and [dema] [TfO] by Yuxiao et al. [29]. A proton conductivity of 0.02 mS/cm was reported at 220 °C. Leaching tests showed that the sulfonic groups from SPEEK and the introduction of silica both helped reduce IL leaking. Specifically, the reduced leaking effect of silica was attributed to electrostatic interactions between the silanol group (Si-OH) and the IL cation.

Our study explores the incorporation of BMI.TfO and hydrophobic PDMS silica (SiO_2_) into polysulfone (PSF) membranes. Polysulfone was chosen for its thermal properties, chemical stability, and mechanical robustness, which are essential for the durability of PEMs. PSF-based membranes have been applied for different applications [30], such as gas separation [31,32], PEM water electrolyzers [33], or PEM-FC [34]. BMI.TfO was selected for its hydrophobic nature and its proven ability to facilitate proton conduction in polymer matrices when used as an additive. The addition of SiO_2_-PDMS aims to enhance IL retention and reduce leakage, thereby improving the operational stability of the membrane. The resulting membranes were thoroughly characterized to understand their morphology, proton conductivity, thermal properties, and IL retention properties. This research aims to provide critical insights into the potential of these modified polysulfone membranes for use in PEMFCs, contributing to the advancement of cleaner and more efficient energy conversion technologies.

## 2. Materials and Methods

### 2.1. Materials

The reagents were used without further purification. Sodium chloride, (NaCl), potas-sium chloride (KCl), and lithium chloride (LiCl), for analysis, as well as polysulfone (average MW~35,000), were purchased from Merck (Rahway, NJ, USA). Sulfuric acid (H_2_SO_4_) at 95–98% and of synthesis grade was purchased from Fisher Chemical (Hampton, NH, USA); 1-n-Butyl-3-methylimidazolium trifluoromethanesulfonate (BMI.TfO) was purchased from Thermo Scientific Alfa Aesar (Haverhill, MA, USA). Chloroform (99.0–99.6% purity) was purchased from VWR International (Radnor, PA, USA), and silicon dioxide (SiO_2_) nanopowder modified by polydimethylsiloxane (PDMS) was purchased from PlasmaChem (Berlin, Germany).

### 2.2. Membrane Preparation

All membranes were prepared by the phase inversion method in ambient conditions, specifically using the dry-casting method [35]. The proper amount of polymer in chloroform and mixed with additives was as follows: (a) SiO_2_ (1 wt% is the maximum solubility in chloroform) and (b) BMI.TfO, if added. As an example, the preparation conditions for PSF_SiO_2__BMI.TfO10% membranes are described as follows: 0.2 g of SiO_2_-PDMS was dissolved in 11.7 mL (17.44 g) of chloroform, with small portions added gradually to facilitate dissolution. Subsequently, 2.36 g of polysulfone was also added in portions. Finally, 2 g of BMI.TfO was introduced. The resulting solution was stirred for 48 h and left overnight for degasification. The obtained mixture was cast on a glass with the use of casting knife (knife thickness: 100 μm) and left under a fume hood for evaporation. The characteristics of each membrane preparation process condition are given in Table 1.

### 2.3. Characterization Methods

#### 2.3.1. Functional Properties

##### Proton Conductivity

Membrane proton conductivity was measured by electrochemical impedance spectroscopy using Metrohm Autolab B.V. in 0.5 M sulfuric acid at room temperature [36]. The EIS measurements were performed with a potential perturbation of 10 mV.rms, and the frequency range was 0.1 Hz–1 MHz. For conductivity in H_2_SO_4_, the membranes were cut into 1 × 1 cm^2^ pieces and soaked in a 0.5 M H_2_SO_4_ solution for 24 h before the test. Additionally, leaking was performed in the H_2_SO_4_ solution of 0.5 M to correlate the conductivity with the real amount of IL in the membrane.

**Figure 1:**
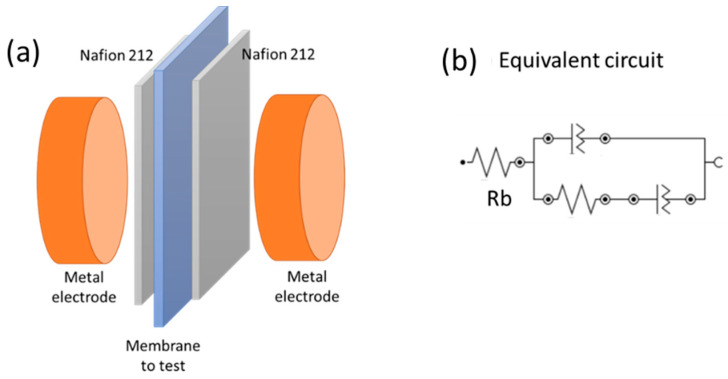
Proton conductivity: (**a**) schematic representation of the cell and (**b**) equivalent circuit.

Samples were sandwiched between two Nafion 212 membranes (used as the blank) and two electrodes (composed of Au-coated Cu) installed in a Teflon holder, corresponding to the through-plane conductivity cell configuration shown in Figure 1.

To ensure perfect contact between the film and the metal electrodes, avoiding impedance noise in the Nyquist plot, a weight of 1.5 kg was added on top of the cell.

The high-frequency data of the Nyquist plot correspond to the combination of bulk resistance (Rb) and capacitance of the polymeric film−electrode system and the fitting of experimental results with the electrical equivalent circuit.

Herein,

R_b_ = R_2Nafion_ + R_membrane to test_

The measurements were carried out three times with three different samples of each membrane, and the average values were reported. The proton conductivity was calculated using the following equation:

(1) $$\sigma =\frac{d}{R\times A}$$

where σ is the conductivity (S/cm), d is the thickness of the wet membrane (cm), R is the ohmic resistance (Ω), and A (=0.25 cm^2^) is the electrode area (cm^2^).

##### Proton Permeability

The proton permeability test was conducted using a Teflon H-cell, where the feed and stripping compartments were separated by the tested membrane. Each compartment held 200 mL of solution. The feed solution consisted of 0.1 M HCl, while the stripping solution contained 0.1 M of the corresponding salt with different cations (LiCl, NaCl, or KCl) to evaluate the influence of the cation size on proton transport. The membrane was cut into 1 × 1 cm pieces and tested without prior pre-treatment. The active membrane area into the cell was 0.66 cm^2^. The experiments were performed over 24 h at ambient temperature and pressure.

At the start of the experiment, the initial pH of the feed solution was recorded. The pH of the stripping solution was continuously measured every 30 s using an Orion 4 Star PH/ISE Multimeter, which was connected to a computer via Orion^TM^ DualStar Com 1.07 software for data collection.

Permeability, *P* [cm^2^/s], was calculated using the following equation:

(2) P=pl

where *p* is the permeability coefficient [cm/s] and *l* is the membrane thickness [cm].

The permeability coefficient was determined based on pH measurements using the following equation:

(3) $$-ln\frac{C_{f}}{C_{0}}\;=\;\frac{Ap}{V_{f}}t$$

where *C*_0_ is the initial proton concentration in the feed solution [mol/L] and *C_f_* is the proton concentration [mol/L] in the feed at time *t* [s].

*C_f_* was calculated from the proton concentration in the stripping solution (*C_s_* [mol/L]) as follows:

(4) $$C_{f}\;={\;C}_{0}-C_{s}$$

Here, *V_f_* [cm^3^] is the feed solution volume and *A* [cm^2^] represents the active membrane area.

##### Ionic Liquid Leaking

The leakage of IL from the membrane was studied in Milli-Q water. Membrane samples of 2 × 2 cm were cut, dried at 60 °C for 72 h, and weighed (*W_i_* [mg]). The dry samples were then immersed in Milli-Q water at room temperature for one week to assess the stability of IL incorporation, and then were separately immersed in the acid solution for 24 h to determine how much IL remained in the membrane after the conditioning for the proton conductivity studies. After the immersion periods, the samples were dried again at 60 °C for 72 h and reweighed (*W_f_* [mg]).

The results are presented as the amount of IL retained in the membrane, considering the initial theoretical IL amount calculated based on the composition of the polymeric solution used for membrane fabrication (Table 1). The decrease (*D* [%]) in IL content was calculated using the following equation:

(5) $$D\;=\;\frac{W_{i}-W_{f}}{W_{i}}100\%$$

It was assumed that any weight loss was solely due to IL leaking.

#### 2.3.2. Thermal Characterization

##### Differential Scanning Calorimetry (DSC)

The determination of melting temperatures (*T*_m_) and glass transition temperatures (*T*_g_) was performed by differential scanning calorimetry (DSC) using a Mettler-Toledo DSC3+ instrument (Columbus, OH, USA) calibrated using indium (heat flow calibration) and zinc (temperature calibration) standards. Samples of approximately 8−10 mg were placed in aluminum pans with pierced lids and analyzed under a flow of N_2_ at 50 mL·min^−1^. The *T*_g_ values of the samples were determined in dynamic scans at 10 °C·min^−1^ from −20 to 250 °C.

##### Thermogravimetric Analysis (TGA)

The thermal stability of the samples was studied by thermogravimetric analysis (TGA) using a Mettler-Toledo TGA 2 thermobalance (Columbus, OH, USA). All the experiments were performed under a flow of N_2_ at 50 mL·min^−1^. Samples of a mass of approximately 10 mg were degraded between 30 and 800 °C at a heating rate of 10 °C·min^−1^.

#### 2.3.3. Morphological and Surface Characterization

##### Environmental Scanning Electronic Microscopy (ESEM)–Energy-Dispersive X-Ray Spectroscopy (EDX)

ESEM-EDX was conducted in a Quanta 600 from FEI Company (Columbus, OH, USA) with an EDX detector managed by Inca Oxford installed in the same equipment. It was used to examine the membranes’ surface morphology and porosity in low-vacuum mode. EDX analysis quantified the elemental composition, confirming the incorporation and distribution of additives, such as ionic liquids or SiO_2_-PDMS. The micrographs were taken by using a magnification value of 1000.

##### Field Emission Scanning Electron Microscopy (FESEM)

FESEM was conducted in a Scios 2 Hi Vac from Thermofisher Scientific (Waltham, MA, USA). It provided high-resolution images of the cross-section, examining morphology and porosity. The samples were immersed into liquid nitrogen and fractured for cross-sectional images. The membrane cross-section micrographs were taken by using a magnification value of 1500.

Images obtained from ESEM and FESEM were further analyzed with ImageJ 153 software in order to obtain information about membrane pore size and thickness.

##### Contact Angle (CA)

Contact angle measurements were performed to determine the hydrophobicity/hydrophilicity and wetting properties of the prepared membranes [37]. The measurements were performed employing Dataphysics OCA 15EC (Dataphysics, Fildertstadt, Germany). A 3 μL droplet of Milli-Q water was placed on to the surface of the membrane. The contact angle was calculated from a digital image by SCA software included in the apparatus. The measurements were repeated three times, and the final result displayed was the average value.

##### Atomic Force Microscopy (AFM)

AFM was applied to supplement morphological and structural features of polymer surfaces [38]. The membrane surface roughness was analyzed by atomic force microscopy (AFM, Molecular Imaging model Pico SPM II (Pico+)) (Scientech, Palaiseau, France), together with Gwyddion 2.61 software. A 50 μm × 50 μm membrane surface area was scanned, and the surface roughness parameters of the membranes were obtained. The average roughness (Ra) is the mean absolute deviation from the mean sample height and the root mean square of the Z data (Rq), and the skewness (Sk) represents the deviation of the pixel height distribution in the AFM image from a Gaussian distribution. A negative value of Sk indicates that the surface is made up of valleys, whereas a surface with a positive skewness is said to contain mainly peaks and asperities.

#### 2.3.4. Chemical Characterization

##### Fourier Transform Infrared Spectroscopy–Attenuated Total Reflectance (FTIR–ATR)

Fourier transform infrared spectroscopy (FT-IR) analysis was performed using a Bruker Vertex 70 instrument (Billerica, MA, USA) with an attenuated total reflectance (ATR) sample holder by acquiring 16 cumulative scans with a 4 cm^−1^ resolution from 4000 to 400 cm^−1^. It was used to identify functional groups and confirm the chemical integrity of the membrane after additive incorporation. 

## 3. Results and Discussion

### 3.1. Functional Properties

Proton Conductivity

The proton conductivity test was performed in H_2_SO_4_ 0.5 M (25 °C), and before the measurements, the membranes were placed in the electrolyte for 24 h for conditioning. To determine the amount of IL in the membranes during the conductivity test, an additional leakage test was performed considering the conditions mentioned above. As described before, the results (Table 2) from IL leaking tests showed that membranes made of polymeric solutions containing 1% of IL did not lose any IL from their structure. However, the membranes prepared out of polymeric solutions with higher amounts of IL, at 5 and 10%, lost 50 and 75% and a similar final amount of IL remained in their structure.

**Figure 5:**
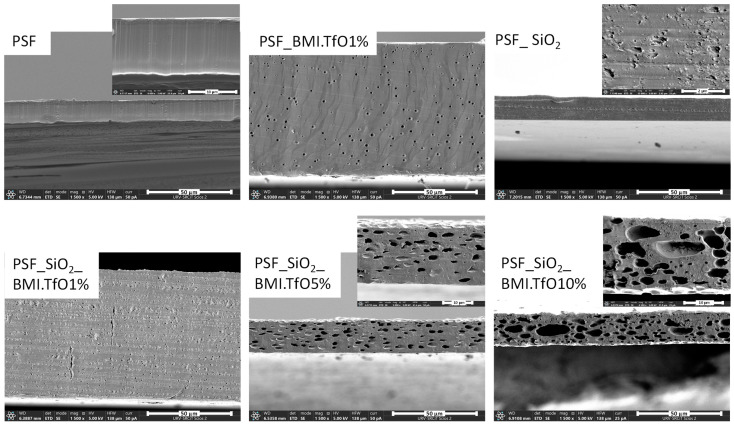
FESEM cross-section pictures. All micrographs are shown with a scale of 50 µm; the inset scale for the thinner membranes (PSF, PSF_SiO_2__BMI.TfO5% and PSF_SiO_2__BMI.TfO10%) is 10 µm and for PSF_SiO_2_, it is 2 µm.

**Figure 6:**
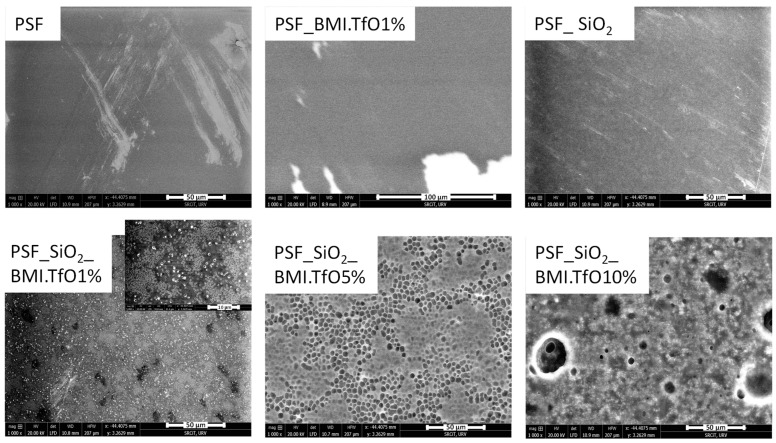
Surface ESEM pictures. All micrographs are shown with a scale of 50 µm, except for PSF_BMI.TfO1% with 100 µm; the inset scale for PSF_SiO_2__BMI.TfO1% is 10 µm.

The conductivity studies showed a positive influence of IL incorporation into the membranes at the highest studied concentrations (Table 2). The blank polysulfone membrane gives a negligibly low value of conductivity, 0.12 [mS/cm], as in the cases of PSF_SiO_2_ and membranes containing 7–8% of IL (PSF_BMI.TfO1% and PSF_SiO_2__BMI.TfO1%), where the proton conductivity was not detected. However, membranes with ~11% of IL and SiO_2_ (PSF_SiO_2__BMI.TfO5% and PSF_SiO_2__BMI.TfO10%) gave values 30× higher than pure PSF, being 2.98 and 3.63 [mS/cm], respectively. We hypothesize that these differences can be attributed to membrane density. As described by Hendrana et al. [39], membranes synthesized from sulfonated polystyrene (sPS) and polyethylene-graft-maleic anhydride (PE-g-MAH) with varying degrees of sulfonation were subsequently compressed to reduce their density. It was demonstrated that decreasing membrane density negatively affected ionic conductivity. In our study, membranes PSF_BMI.TfO1% and PSF_SiO_2__BMI.TfO1% are significantly denser compared to PSF_SiO_2__BMI.TfO5% and PSF_SiO_2__BMI.TfO10%, as described in Section 3.3 (Morphological and Surface Properties). This difference in density likely explains the large differences in proton conductivity, despite having similar IL content (8–7 wt% vs. 11.5–11 wt%). A similar explanation applies to the differences in conductivity between PSF_SiO_2__BMI.TfO5% and PSF_SiO_2__BMI.TfO10%, where larger pores are observed in both cross-sectional and surface micrographs (Figures 5 and 6). For reference, a Nafion membrane with a typical dry thickness of 50.8 µm [40] tested under the same conditions showed a proton conductivity of 17.98 mS/cm.

Proton Permeability

In this study, proton permeability measurements were performed using Li^+^, Na^+^, and K^+^ counter-cations in the stripping solution to assess the influence of counter-ion size on proton transport. However, no direct correlation between proton permeability and cation size was observed, indicating that proton transport through the membrane is not significantly affected by the counter-ion size in this system.

The results (Table 3) show that the base PSF membrane and PSF-SiO_2_ membranes, along with those containing low amounts of IL (1%), exhibited extremely low proton permeability, with some values being undetectable. These results suggest that without sufficient IL incorporation, the membranes lack the necessary pathways for effective proton transport.

In contrast, membranes with higher IL concentrations (PSF-SiO_2_-BMI.TfO5% and PSF-SiO_2_-BMI.TfO10%) demonstrated up to a five-fold improved proton permeability, reaching values of up to 3.5 · 10^−7^ cm^2^/s. This indicates that the presence of IL in higher amounts enhances the proton transport properties of the membrane, likely by creating more favorable ionic pathways for proton conduction. The increase in permeability with higher IL content aligns with the previous observations regarding the structural changes in the membrane, including increased porosity and the formation of more interconnected channels, which facilitate proton migration. Those permeability results were consistent with the previously discussed proton conductivity. However, even with the improved performance of the high-IL membranes, their proton permeability remains lower than that of the benchmark Nafion membrane, which exhibits permeability values one order of magnitude higher, in the range of 10^−6^ cm^2^/s.

Ionic Liquid Leaking Tests

The leakage of IL from the membranes was studied in Milli-Q water to assess the stability of the IL within the polymer matrix and the impact of membrane composition on IL retention. Ensuring minimal IL leakage is critical for maintaining the ionic conductivity and durability of the membranes in fuel cell applications, where prolonged exposure to humid environments is expected.

**Figure 2:**
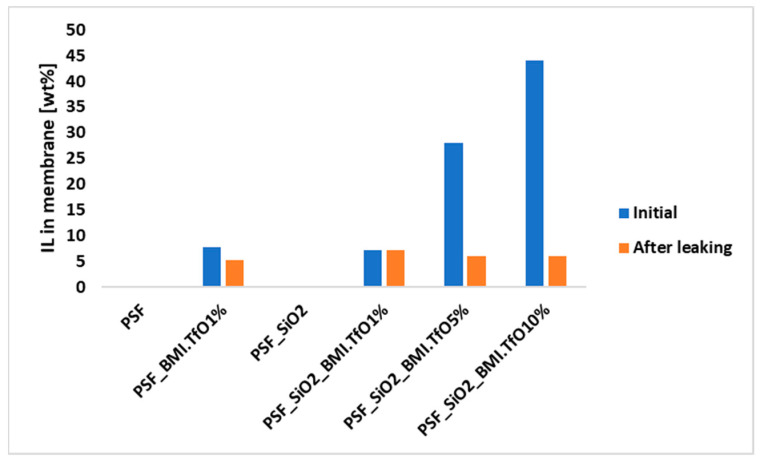
IL leakage.

When the membrane was prepared with PSF and 1% IL, a leakage of 32% (from 8% to 5% of IL content) was observed after one week in water, despite the fact that both materials are water-insoluble (Figure 2). However, when hydrophobic silica (SiO_2_-PDMS) was incorporated into the polymer matrix, no leakage of IL was detected. This suggests that SiO_2_ plays a critical role in stabilizing the membrane structure and retaining IL, making the membrane more suitable for fuel cell applications by preventing the loss of key functional components.

As the IL content was increased into the membrane to 28 wt% and 44 wt% (solutions of 5% and 10%, respectively), the leakage significantly increased to 79 and 86%, respectively. This increase is likely due to the larger pore sizes in these membranes (Table 5), which facilitate greater IL migration out of the membrane. The relationship between pore size and leakage is further supported by the contact angle measurements, where higher contact angles (Table 6), indicating greater hydrophobic surface, correlated with lower leakage values.

After the leakage tests, the remaining IL content in all membranes was found to be similar, ranging from 5.3 to 7.2 wt%, regardless of the initial concentration. This suggests a limit for IL retention within the membrane, which may serve as a limiting factor for its performance in PEM fuel cells.

### 3.2. Thermal Characterization and Thermal Stability

**Figure 3:**
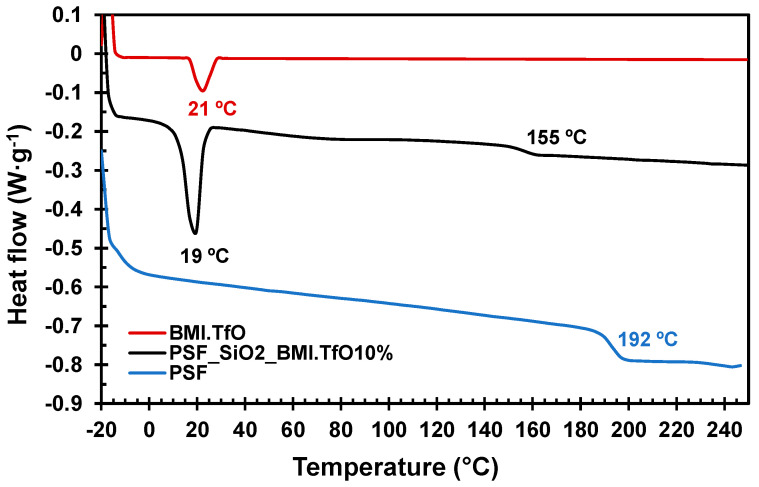
DSC thermogram of the BMI.TfO (red), PSF_SiO_2__BMI.TfO10% membrane (black), and the neat PSF (blue) from −20 °C to 250 °C.

With the aim of demonstrating the viability of the prepared membranes in high-temperature fuel cell applications, the membrane was thermally characterized and compared with both the neat polysulfone and the ionic liquid. The DSC thermogram (Figure 3) shows that the membrane (black line) presents a *T*_g_ of 155 °C, which is considerably lower than the neat PSF (blue line) (192 °C). This fact can be attributed to the presence of the ionic liquid that acts as a plasticizer. A *T*_m_ at 19 °C can also be observed, which correlates to the melting temperature of the ionic liquid (red line) (21 °C). The presence of this *T*_m_ corroborates the correct inclusion of the ionic liquid into the polymeric membrane. Although the decrease in the *T*_g_ is noteworthy, compared to the original PSF, it demonstrated that this membrane can be used up to 140 °C without a significant decrease in its physical properties. This calorimetric analysis reveals that the prepared membrane has a higher *T*_g_ than that reported for Nafion^®^ membranes (132 °C) [41]. Table 4 collects all the calorimetric data of the samples, as well as the thermal stability determined by TGA.

**Figure 4:**
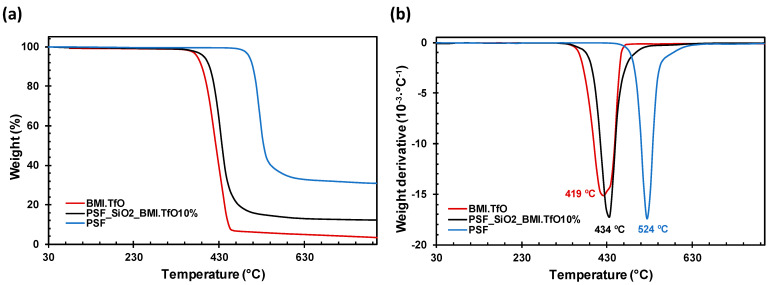
(**a**) Superposed TGA thermograms and (**b**) superposed TGA 1st derivative of the BMI.TfO (red), PSF_SiO_2__BMI.TfO10% (black), and PSF (blue).

The thermal stability was studied with thermogravimetric analysis (TGA) (Figure 4). Interestingly, the temperature of the maximum rate of degradation of the membrane (PSF_SiO_2__BMI.TfO10%) is considerably lower (434 °C) than that of the neat PSF (524 °C). We hypothesize that the ionic liquid, or its degradation products, could act as a catalyst in the thermal decomposition of the polysulfone, as the *T*_max_ of the ionic liquid is just below that of the membrane (419 °C). It is reported in the literature that there is a lower thermal stability of polysulfone in the presence of ionic liquids [31]. Nevertheless, the *T*_2%_ of the membrane (367 °C) is far above any possible temperature applications, showing that the membrane will be stable for a long period at high temperatures. The char yield of PSF_SiO_2__BMI.TfO10% (12.0%) is between the neat PSF (30.7%) and the ionic liquid (2.4%), which is another piece of evidence of the correct incorporation of the ionic liquid into the polymeric matrix and that a considerable fraction of the membrane is ionic liquid content.

### 3.3. Morphological and Surface Properties

The surface and cross-section morphology of the membranes were examined using SEM, and the corresponding images are displayed in Figure 5 and Figure 6. The micrographs show significant differences in pore structure and distribution between the membranes with and without additives (see Table 5 for details on membrane thickness, porosity, and pore size distribution).

In the solvent evaporation process used for membrane fabrication, a polymer solution is cast on a glass plate in the form of a thin film, and the solvent is gradually evaporated, resulting in a solidified membrane structure. This process, a variant of phase inversion known as dry-casting, relies on physical phase transitions. The composition of the polymeric solution—including any additives—affects the membrane’s final properties, such as porosity, pore size distribution, and structural morphology [35].

Additives play a significant role in modifying the thermodynamic interactions within the solvent–polymer system, thus influencing the phase separation and polymer chain dynamics. For instance, a slower evaporation rate allows for more time for polymer chain rearrangement, leading to a more compact and denser membrane structure. In contrast, a rapid solvent evaporation promotes phase separation, resulting in increased porosity and a more open, interconnected pore structure.

For the pure polysulfone (PSF) membrane, the SEM images reveal a relatively compact and smooth structure, with limited pore formation, as is typically expected for membranes prepared from polymers without additives using the dry-casting method [35]. The incorporation of SiO_2_ in the PSF membrane matrix promotes the formation of irregular pores (0.15 ± 0.10 µm in the cross-section).

The PSF_SiO_2_ membrane shows a more open and porous structure compared to the pure PSF, suggesting that the silica particles act as pore formers during the membrane fabrication process.

The addition of IL alone (PSF_BMI.TfO1%) also leads to pore formation (1.3 ± 0.32 µm in the cross-section), but the morphology is distinct from that of the SiO_2_-containing membranes. The cross-sectional images of PSF_BMI.TfO1% show larger and more circular but fewer pores compared to PSF_SiO_2_, indicating that the IL promotes a different pore-forming mechanism.

When both SiO_2_ and IL are combined (PSF_SiO_2__BMI.TfO1%, PSF_SiO_2__BMI.TfO5%, and PSF_SiO_2__BMI.TfO10%), the resulting membranes exhibit the most significant morphological changes. PSF_SiO_2__BMI.TfO 1% shows moderate pore sizes (0.58 ± 0.16 µm in the cross-section and 0.5 ± 0.10 µm on the surface). However, as the IL content increases to 5% and 10%, a highly porous structure with well-defined, interconnected pores was obtained. PSF_SiO_2__BMI.TfO 5% displays a cross-sectional pore size of 2.6 ± 1.5 µm and surface pore size of 4.0 ± 1.0 µm, while PSF_SiO_2__BMI.TfO10% shows an even larger cross-sectional pore size (4.2 ± 2.5 µm) and surface pore size of 17 ± 5.0 µm.

In terms of surface morphology, SEM images reveal a clear trend; the surface porosity increases with higher concentrations of IL and SiO_2_. The surface of the pure PSF membrane appears smooth, lacking significant surface features or pores, as well as membranes with only one of the additives (SiO_2_ or 1% IL). However, with the introduction of both additives, the surface becomes increasingly textured, with visible pores appearing across the membrane surface.

It is noteworthy that the PSF_SiO_2__BMI.TfO10% membrane, which contains the highest concentration of IL (10%), exhibits the most pronounced surface roughness and porosity. The presence of large open pores on the surface of this membrane suggests that the additive combination not only affects the internal pore structure but also influences the external surface characteristics. This surface roughness is likely to impact the membrane’s interaction with liquids, which is further discussed in the contact angle analysis.

The membrane thickness, which is influenced by the presence of additives, also plays a critical role in overall membrane performance. The SEM cross-sectional images show that membranes containing 1% IL (PSF_BMI.TfO1% and PSF_SiO_2__BMI.TfO1%, translating to 8 and 7 wt% for IL content in the final PEM) are significantly thicker (78 ± 0.50 µm and 75 ± 0.69 µm, respectively) than the pure PSF membrane (12 ± 0.30 µm). The increased thickness could be attributed to the plasticizing effect of IL, which facilitates the formation of a more expanded structure during the casting method. However, as the IL content increases, such as in PSF_SiO_2__BMI.TfO5% (28 wt%) and PSF_SiO_2__BMI.TfO10% (44 wt%), the membrane thickness decreases to 18 ± 0.69 µm and 19 ± 1.5 µm, respectively. This reduction in thickness is likely due to the increased viscosity of the casting solution. This indicates that while the additives enhance porosity, they also affect membrane compaction and mechanical integrity, which is important for its practical application. Additionally, the plasticizer effect of IL on membrane degradation was demonstrated in Section 3.2 though DSC analysis. Farrokhara et al. investigated IL-modified PSF membranes with varying IL content for gas separation applications. They demonstrated that incorporating IL as a plasticizer into the polymer matrix reduces viscosity, leading to more porous and irregular structures. Additionally, they established a correlation between higher IL content and the formation of larger pores [31].

**Table 5:** Membrane characteristics.

Membrane	IL in Membrane (Theor.)	Cross-Section (µm)		Surface Pore Size (µm)
		Thickness	Pore Size	
PSF	-	12 ± 0.30	-	-
PSF_BMI.TfO1%	8 wt%	78 ± 0.50	1.3 ± 0.32	-
PSF_SiO_2_	-	13 ± 0.72	0.15 ± 0.10	-
PSF_SiO_2__BMI.TfO1%	7 wt%	75 ± 0.69	0.58 ± 0.16	0.5 ± 0.10
PSF_SiO_2__BMI.TfO5%	28 wt%	18 ± 0.69	2.6 ± 1.5	4 ± 1.0
PSF_SiO_2__BMI.TfO10%	44 wt%	19 ± 1.5	4.2 ± 2.5	17 ± 5.0

As presented in Table 5, the pure polysulfone (PSF) membrane surface displays a slightly hydrophilic nature with a contact angle of 79 ± 2°. However, the addition of SiO_2_ or ionic liquid (IL) alters the surface wettability significantly. Although the hydrophobic nature of both additives led to the expectation of increased membrane hydrophobicity, the wettability is influenced by both the material composition and the resulting surface structure [37]. Determining the dominant factor between these two effects is challenging, as both SiO_2_ and IL independently slightly increase contact angle values. Nonetheless, the contact angles remain below 90°, indicating that the modified membranes continue to exhibit hydrophilic characteristics.

When SiO_2_ and 1% IL are combined, the contact angle surpasses 100°, resulting in a transition to a hydrophobic surface. Interestingly, increasing the IL content beyond 1% reverses this trend, causing the contact angle to decrease. As it can be seen in Table 6, when the IL concentration is increased to 5% and 10%, the roughness escalates dramatically (Ra = 329.9 nm and 453.5 nm, respectively) but the contact angle decreases (80° for 5% IL and 75° for 10% IL). This unexpected behavior can be attributed to excessive roughness leading to heterogeneous wetting, where water partially penetrates the membrane’s porous structure, diminishing its hydrophobicity.

**Figure 7:**
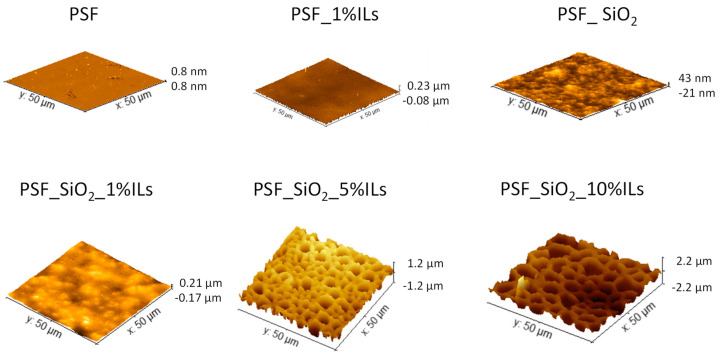
AFM height images obtained for the membrane surfaces.

Table 6 shows the average roughness (Ra), root mean square roughness (Rq), and skewness (Sk) data obtained from atomic force microscopy (AFM), and images of surface roughness obtained from Gwyddion 2.61 software are displayed in Figure 7.

**Table 6:** Contact angle values and numerical roughness data.

Membrane	Contact Angle [°]	Average Roughness (Ra) [nm]	Root Mean Square (Rq) [nm]	Skewness (Sk)
PSF	79 ± 2	0.7	1.6	−4.7
PSF_BMI.TfO1%	87 ± 2	17.9	21.8	−0.64
PSF_SiO_2_	87 ± 1	4.4	5.7	1.1
PSF_SiO_2__BMI.TfO1%	102 ± 4	52.8	64.0	−0.53
PSF_SiO_2__BMI.TfO5%	80 ± 2	329.9	395.1	−0.45
PSF_SiO_2__BMI.TfO10%	75 ± 2	453.5	539.4	−0.12

The AFM roughness data align well with the surface roughening observed in the SEM micrographs, increasing significantly as the concentration of additives rises.

### 3.4. Chemical Characterization

**Figure 8:**
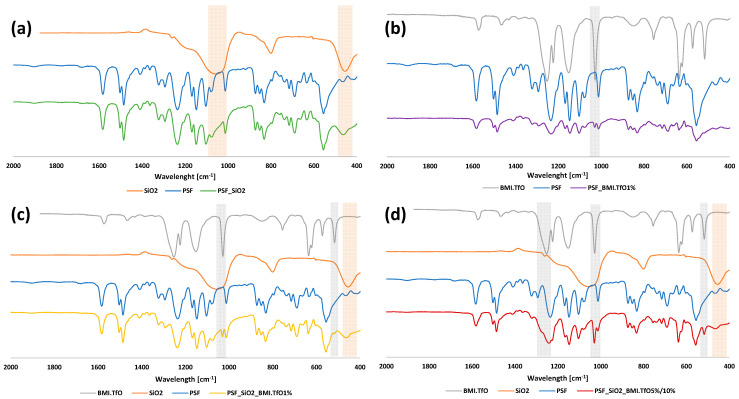
FTIR-ATR spectra of the membranes. (**a**) PSF_SiO_2_, (**b**) PSF_BMI.TfO1%, (**c**) PSF_SiO_2__BMI.TfO1%, and (**d**) PSF_SiO_2__BMI.TfO5%/10%.

The FTIR spectra (Figure 8) confirm the successful incorporation of SiO_2_ and IL into the PSF membranes by identifying characteristic peaks corresponding to each additive. In PSF_SiO_2_, SiO_2_ is detected at 1054 cm^−1^ (broadening of the C-O stretch) and at 455 cm^−1^ (new Si-O peak), indicating its interaction with the PSF matrix.

For PSF_BMI.TfO1%, the IL is detected at 1030 cm^−1^ with a new S=O peak. Although PSF itself has an S=O stretch, the 1% IL membrane shows an intensified peak at this wavelength, suggesting the contribution of the IL to the overall S=O signal.

In the membranes containing both SiO_2_ and IL (1%, 5%, and 10% IL), the same SiO_2_ peaks at 1054 cm^−1^ and 455 cm^−1^ are observed, alongside the IL peak at 1030 cm^−1^. At higher IL concentrations (5% and 10%), additional peaks emerge at 514 cm^−1^ (C-F) and 1272 cm^−1^ (C-O), indicating more pronounced interactions between the IL and the PSF matrix. These new functional groups reflect the structural changes in the polymer network, likely contributing to the membrane’s altered properties, such as increased porosity.

Elemental analysis from ESEM-EDX (see Table 7) confirms the presence of carbon, oxygen, and sulfur present in polysulfone in all membranes, with silicon (from SiO_2_–PDMS) and fluorine (from IL) detected in the appropriate samples. Trace amounts of chlorine were found in almost all membranes, likely originating from residual chloroform used as a solvent during membrane preparation.

## 4. Conclusions

Polysulfone-based membranes modified with ionic liquid (IL) and PDMS-functionalized silica (SiO_2_-PDMS) were prepared and characterized as prospective proton exchange membranes (PEMs) for fuel cells operating at middle and elevated temperatures. The addition of IL and SiO_2_-PDMS resulted in significant internal and external structural modifications, increasing both the porosity and surface roughness of the membranes. The incorporation of SiO_2_-PDMS helped reduce IL leakage, particularly in membranes with lower IL content (1%). However, membranes with higher IL content (5% and 10%) exhibited increased leakage due to larger and interconnected pores, suggesting a limit for IL retention within the membrane.

Membranes with 5% and 10% of IL concentration in the polymeric solution showed promising results in terms of proton conductivity and permeability, enhancing polysulfone efficiency by 30 times and 4–5 orders of magnitude, respectively. This improvement was attributed to the membrane density, where higher porosity may create more accessible ionic pathways.

EDX analyses and FTIR spectra prove the existence of IL and SiO_2_-PDMS in the membranes. Thermal characterization indicated that the modified membranes maintained stability at elevated temperatures, with thermal degradation temperatures exceeding typical PEMFC operating conditions, hence being compatible with such applications. The introduction of IL into the PSF matrix lowered its glass transition temperature (*T*_g_) as expected, but the membranes remained usable up to 140 °C, balancing thermal stability with proton conductivity, which is crucial for long-term fuel cell operation.

While the results are promising, further studies are needed to better understand the long-term stability and possible means of optimization of these membranes. The relationship between IL content, membrane porosity, and IL retention must be further explored to enhance membrane durability and efficiency for practical PEMFC applications. In conclusion, the incorporation of IL and SiO_2_-PDMS into the polysulfone matrix enhances the properties of proton exchange membranes, showing great potential for PEMFC applications. In particular, the use of PSF–ionic liquid membranes offers a promising perspective beyond Nafion, primarily due to their thermal stability, connected to its ability to operate at higher temperatures and its reduced dependency on hydration for proton transport. However, further investigations are necessary to validate their long-term stability and practical feasibility.

## Figures and Tables

**Table 1:** Polymeric solution composition used for preparation of membranes.

Membrane	PSF (wt%)	Chloroform (wt%)	PDMS-SiO_2_ (wt%)	BMI.TfO (wt%)
PSF	12	88	-	-
PSF_BMI.TfO1%	12	87	-	1
PSF_SiO_2_	12	87	1	-
PSF_SiO_2__BMI.TfO1%	12	86	1	1
PSF_SiO_2__BMI.TfO5%	11	83	1	5
PSF_SiO_2__BMI.TfO10%	11	78	1	10

* n.d., not detected.

* n.d., not detected.

**Table 2:** Proton conductivity.

Membrane	Average Wet Thickness [µm]	IL in Membrane After 24 h in Electrolyte [%]	Proton Conductivity [mS/cm] in 0.5 M H_2_SO_4_ (RT)
PSF	13 ± 0.0	-	0.12 ± 0.01
PSF_BMI.TfO1%	84 ± 3.7	8 wt%	n.d. *
PSF_SiO_2_	20 ± 1.2	-	n.d. *
PSF_SiO_2__BMI.TfO1%	72 ± 1.7	7 wt%	n.d. *
PSF_SiO_2__BMI.TfO5%	27 ± 0.5	11.5 wt%	2.98 ± 0.068
PSF_SiO_2__BMI.TfO10%	23 ± 0.0	11 wt%	3.63 ± 0.275
Nafion	66 ± 0.0	-	18.0 ± 0.16

* n.d., not detected.

**Table 3:** Proton permeability results.

Membrane	Proton Permeability [cm^2^/s]		
	Li^+^	Na^+^	K^+^
PSF	1.2 · 10^−11^	1.2 · 10^−10^	n.d.
PSF_BMI.TfO1%	2.4 · 10^−11^	n.d. *	3.6 · 10^−12^
PSF_SiO_2_	5.1 · 10^−11^	n.d. *	7.7 · 10^−11^
PSF_SiO_2__BMI.TfO1%	2.4 · 10^−11^	n.d. *	n.d. *
PSF_SiO_2__BMI.TfO5%	2.4 · 10^−7^	1.6 · 10^−7^	2.4 · 10^−7^
PSF_SiO_2__BMI.TfO10%	2.1 · 10^−7^	1.4 · 10^−7^	3.5 · 10^−7^
Nafion	4.2 · 10^−6^	5.9 · 10^−6^	3.5 · 10^−6^

* n.d., not detected.

**Table 4:** Calorimetric data and thermal stability data of the studied samples under N_2_.

Sample	*T*_m_ ^a^ (°C)	*T*_g_ ^b^ (°C)	*T*_2%_ ^c^ (°C)	*T*_max_ ^d^ (°C)	Char Yield ^e^ (%)
BMI.TfO	21	-	357	419	2.4
PSF_SiO_2__BMI.TfO10%	19	155	367	434	12.0
PSF	-	192	488	524	30.7

^a^ The melting temperature of the samples. ^b^ The glass transition temperature of the samples. ^c^ The temperature of 2% of weight loss in N_2_. ^d^ Temperatures at the maximum rate of degradation. ^e^ Char residue at 800 °C.

**Table 7:** Elemental mapping table.

Membrane	Elements (wt%)					
	C	O	S	Si	F	Cl
PSF	78.2	13.2	7.9	n.d.	n.d.	0.70
PSF_BMI.TfO1%	75.7	12.9	7.3	n.d.	1.9	2.2
PSF_SiO_2_	73.8	15.6	6.6	3.6	n.d.	0.4
PSF_SiO_2__BMI.TfO1%	73.1	14.6	7.9	2.3	2.1	1.0
PSF_SiO_2__BMI.TfO5%	68.4	16.0	8.4	2.4	4.8	n.d.
PSF_SiO_2__BMI.TfO10%	66.7	14.2	10.2	2.2	6.5	0.20

n.d., not detected.

## Data Availability

The original contributions presented in the study are included in the article, further inquiries can be directed to the corresponding author.
